# Mutual Effects of Fluorine Dopant and Oxygen Vacancies on Structural and Luminescence Characteristics of F Doped SnO_2_ Nanoparticles

**DOI:** 10.3390/ma10121398

**Published:** 2017-12-07

**Authors:** Xiaolong Wang, Xuan Wang, Qingyin Di, Hongli Zhao, Bo Liang, Jingkai Yang

**Affiliations:** State Key Laboratory of Metastable Materials Science and Technology, College of Materials Science and Engineering, Yanshan University, Qinhuangdao 066004, China; wangxiaolong920304@163.com (X.W.); wangxysu@163.com (X.W.); Q15903393973@163.com (Q.D.); zhaohongli@ysu.edu.cn (H.Z.)

**Keywords:** F doped SnO_2_ nanoparticles, hydrothermal method, photoluminescence property, oxygen vacancies

## Abstract

SnO_2_ and F doped SnO_2_ (FTO) nanoparticles (NPs) have been synthesized by the hydrothermal method with subsequent annealing at 500 °C. The microstructure and photoluminescence (PL) property of SnO_2_ and FTO NPs have been investigated, and an assumption model about the luminescence process of FTO NPs has been proposed. All of the SnO_2_ and FTO NPs possess polycrystalline tetragonal rutile structures, and the average size in the range of 16.5–20.2 nm decreases with the increasing of F doping content. The doping element F is shown a uniformly distribution by electron energy loss spectroscopy (EELS) mapping. The oxygen vacancy concentration becomes higher as is verified by Raman and X-ray photoelectron spectra (XPS). There are three kinds of oxygen chemical states in SnO_2_ and FTO NPs, in which O_α_ corresponds to oxygen vacancies. The room temperature PL position is observed to be independent of F doping content. F^−^ may substitute O^2−^ into the SnO_2_ lattice by generating $F_{O}^{+}$ and one extra *e*^−^, which can combine with $V_{O}^{+}$ or $V_{O}^{++}$ to generate $V_{O}^{0}$ or $V_{O}^{+}$ to ensure charge balance.

## 1. Introduction

Nowadays, great interest has been attracted in the photoluminescence (PL) properties of many wide direct band gap semiconductors, due to their promising applications in short-wavelength optical devices, such as fluorescent lamps, plasma display panels (PDPs), light emitting diodes (LEDs), laser diodes (LDs), and so on [1,2,3,4]. Tin oxide (SnO_2_) is a promising functional n-type semiconductor with a wider band gap (3.6 eV at 300 K), as well as a higher exciton binding energy (130 meV) (when compared to that of ZnO (3.25 eV and 60 meV, respectively)), and has attracted much attention in recent years [5,6,7,8,9].

It is well known that the point defects, such as oxygen vacancies, interstitial ions, or substitutional ions, will play a particularly important role in the luminescence process of SnO_2_ [10,11,12,13,14]. In general, the dopants can obviously improve the property of semiconductor materials by producing the point defects. In order to enhance the PL property of SnO_2_, various cation dopants were introduced into SnO_2_, such as Co^2+^, Fe^3+^, Ni^2+^, Ga^3+^, Sb^5+^, Cr^3^^+^, and so on. Ahmed et al. [15] pointed that the enhancement of the visible emission of Ni doped SnO_2_ nanoparticles could be attributed to the oxygen vacancies that are created by the substitution of Sn^4+^ by Ni^2+^. Pei et al. [16] believed that the origin of the major PL peak was ascribed to the transition between donor levels of *Ga_in_*, oxygen vacancies and acceptor level of *Ga_sub_*. Wang et al. [17] reported that the main origin of the UV-violet luminescence band can be attributed to the electron transition between oxygen vacancies and the acceptor level that is formed by Sb ions. Therefore, it is considered that oxygen vacancies are the most common defects in metal oxides and usually act as radiative centers in luminescence processes.

When compared with the cation dopants, anion doping is another useful method to create oxygen vacancies. The radius of F^−^ ($r_{F^{-}}$ ≈ 1.33 Å) is slightly less than that of O^2−^ ($r_{O^{2-}}$ ≈ 1.40 Å) and electronegativity of F^−^ ($\chi_{F^{-}}$ ≈ 4.368) is more pronounced than O^2−^ ($\chi_{O^{2-}}$ ≈ 3.758). Therefore, it is much easier for F^−^ to replace O^2−^ in the SnO_2_ lattice and generate oxygen vacancies [18,19,20,21,22,23,24]. Shewale et al. [6] obtained an intense violet emission at 404 nm with a shoulder peak at 396 nm of F doped SnO_2_ (FTO) films deposited by advanced spray pyrolysis technique at low substrate temperature. Thirumoorthi et al. [20] excitated the (211) oriented FTO films in the range of 350–550 nm, and obtained a sharp dominant peak at 362 nm with shoulder peak at 377 nm. Zhu et al. [22] recorded the PL spectra of FTO films prepared by RF reactive magnetron sputtering at low substrate temperature, and obtained PL peaks centered at about 375 nm, 405 nm, 440 nm and 530 nm, respectively. Ajili et al. [25] obtained PL peaks of FTO films that were prepared by chemical spray pyrolysis centered at about 372 nm, 486 nm, 530 nm, and 719 nm, respectively. However, there is still controversy with regard to the effects of oxygen vacancies on the PL properties of SnO_2_, and the related mechanisms have remained as an issue of debates.

In general, the PL processes are measured mainly under the 325 nm wavelength excitation [20,21,22,23,24,25]. Actually, the emission wavelength of low pressure mercury vapor is about 254 nm in Fluorescent lamps. The emission wavelength of inert gases in PDP display is below 200 nm. The emission wavelength on UV conversion fluorescent powder in LEDs is in the range of 200–360 nm. Therefore, it is necessary to investigate the PL property of FTO materials under shorter light excitation.

What is more, when compared with FTO films, the reports on PL property of FTO nanoparticles (NPs) are much less. The influences of F doping content on properties of FTO NPs have not been studied, yet. Accordingly, it is meaningful to study the mutual effects of F dopant and oxygen vacancies on structural and luminescence characteristics of FTO NPs.

In the present work, the primary objective is to conduct more precise analysis on PL properties of FTO NPs under shorter light excitation, especially focus on mutual effects of F dopant and oxygen vacancies on PL properties. SnO_2_ and FTO NPs were firstly synthesized by a simple and effective hydrothermal method, with subsequent annealing at 500 °C. X-ray diffraction (XRD), Transmission electron micrograph (TEM) and X-ray photoelectron spectra (XPS) were carried out to investigate the microstructure. The PL performance was conducted under 255 nm wavelength excitation. The mechanism and the position of defect energy level in the band gap are discussed in detail, and an assumption model about the PL process of FTO NPs will be proposed.

## 2. Experimental

In this work, SnO_2_ and FTO NPs were synthesized by hydrothermal process. Tin chloride pentahydrate (SnCl_4_·5H_2_O) and ammonium fluoride (NH_4_F) were used as the starting material and F dopant source, respectively. Based on the references [21,22,24,26,27], the dopant content (at. % F to Sn) was designed as 0 at. %, 10 at. %, 30 at. %, and 50 at. % (NH_4_F: 0.000 g, 0.148 g, 0.444 g, 0.741 g), respectively. All of the raw materials were obtained from Merck, India, were and used without further purification.

Firstly, 14.024 g SnCl_4_·5H_2_O was dissolved into 75 mL deionized water mixed with 75 mL ethanol (C_2_H_5_OH) and 2 mL concentrated hydrochloric acid (HCl). Secondly, quantitative NH_4_F was added to the above solution under stirring. After that, concentrated ammonia (NH_3_·H_2_O) was added into the above solution to adjust pH to 2.0. The mixed solution was stirred magnetically for 4 h to obtain homogenous precursor solution. Thirdly, the mixed solution was transferred into the 200 mL Teflon-sealed autoclave and held at 180 °C for 12 h. After that, the obtained precipitation was filtered and washed with distilled water and alcohol several times to remove impurities completely, then dried at 80 °C for 4 h, and then grounded into powders for annealing. Finally, the powders were annealed at 500 °C for 0.5 h in a muffle furnace to obtain the SnO_2_ and FTO NPs.

The crystal structure of SnO_2_ and FTO NPs was determined by X-ray diffraction (XRD, D/max-2500PC diffractometer, Rigaku, Tokyo, Japan) with Cu-K*α* radiation (*λ* = 0.15406 nm). Selected area electron diffraction (SAED), high resolution transmission electron microscopy (HRTEM) images, as well as electron energy loss spectroscopy (EELS) mapping were performed by transmission electron microscopy (TEM, JEM-2010 spectrometer, JEOL, Tokyo, Japan). The Raman spectroscopy was carried out by a Renishaw2000 invia Raman spectrometer (London, UK). UV–vis spectrometer (UV-3150, Shimadzu, Kyoto, Japan) was taken to record Diffuse reflectance spectra (DRS) using BaSO_4_ as a reference. Chemical composition and the various elements chemical state were investigated by X-ray photoelectron spectroscopy (XPS, ESCALAB-250Xi spectrometer, Thermo Fisher Scientific, Waltham, MA, USA). Al K*α* was used as the excitation source with X-ray spot size of 0.25 mm. PL performance was obtained by a fluorescence spectrophotometer (F-2500 spectrophotometer, Hitachi, Tokyo, Japan) at room temperature.

## 3. Results and Discussion

### 3.1. XRD Analysis

**Figure 1:**
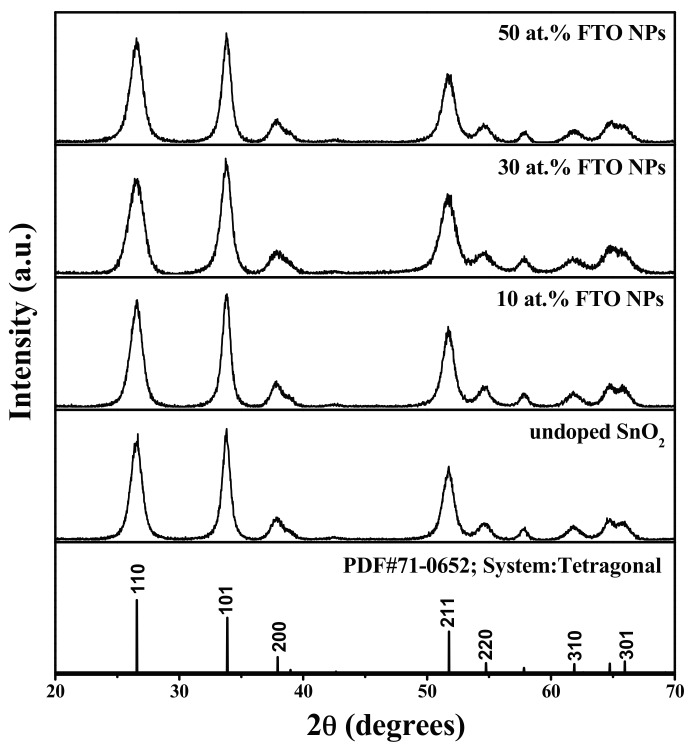
X-ray diffraction (XRD) patterns of tin oxide (SnO_2_) and F doped SnO_2_ (FTO) nanoparticles (NPs).

XRD patterns of SnO_2_ and FTO NPs are shown in Figure 1. All of the diffraction peaks belong to SnO_2_ phase with polycrystalline tetragonal rutile structure (JCPDS No. 71-0652). No other phases corresponding to fluoride are detected, indicating that fluorine ions are incorporated into the SnO_2_ crystal lattice, or that F content is too little to be detected [18,19,20,21,22,23,24].

The crystallite size of (110), (101) and (211) planes of SnO_2_ and FTO NPs was calculated using the Scherrer Equation [27,28,29]:

(1) D=0.9λ/(βcosθ)

where D is the crystallite size, *λ* is X-ray wavelength (0.15406 nm), *θ* is the Bragg diffraction angle, and *β* is the full width at half maximum of the diffraction peak (FWHM). The average crystallite size (D_aver_) of (110), (101), and (211) planes, interplanar spacing (D-spacing), as well as the lattice parameters of SnO_2_ and FTO NPs are listed in Table 1. It can be seen that the average crystalline size, D-spacing of (211), lattice parameter “a”, and cell volume “V” decrease with the increasing of F doping content from 0 at. % to 50 at. %. This can be explained by the incorporation of F^−^ into the SnO_2_ lattice, as well as the increase in the population of oxygen vacancies [18,19]. Because of the slight difference of the ion radius and charge number between O^2−^ and F^−^, F^−^ can substitute O^2−^ to occupy the regular lattice sites in SnO_2_ host. This may lead to the internal stress, charge imbalance, and lattice distortion in the SnO_2_ lattice, ultimately inhibiting the growth of SnO_2_ crystallites [8,15,18]. Moreover, the lattice distortion and charge imbalance may contribute to the formation of point defects (especially oxygen vacancies) [7,8,9], resulting in the decrease of lattice parameters further. Similar discussions have been reported by Kaur et al. [10].

### 3.2. TEM Analysis

**Figure 2:**
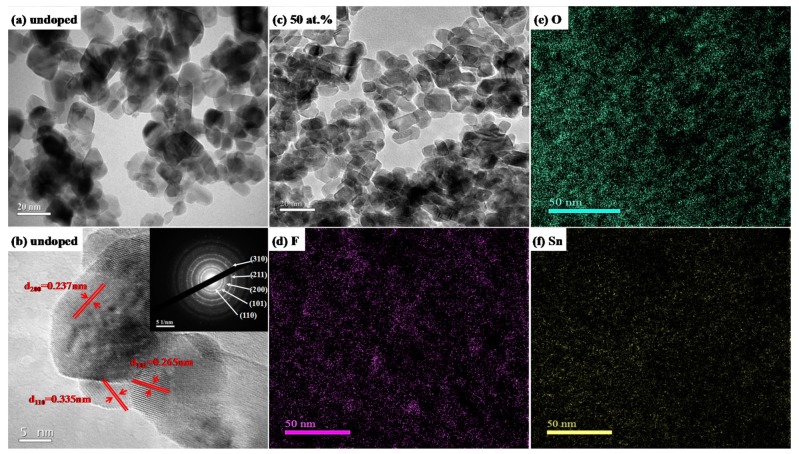
Transmission electron micrograph (TEM) images, high resolution transmission electron microscopy (HRTEM), selected area electron diffraction (SAED) pattern, and electron energy loss spectroscopy (EELS) maps of SnO_2_ and FTO NPs: (**a**) TEM image of SnO_2_ NPs; (**b**) HRTEM image with inset SAED pattern of SnO_2_ NPs; (**c**) TEM image of FTO NPs with 50 at. % F doping; (**d**–**f**) F, O and Sn EELS element mapping of FTO NPs with 50 at. % F doping, respectively.

Figure 2 presents TEM images, SAED pattern, HRTEM images and EELS maps of SnO_2_ and FTO NPs. It is obvious that the obtained particles of SnO_2_ and FTO NPs are spherical and hexagonal shape, as shown in Figure 2a,c. The average grain sizes are about 20 nm and 14 nm for SnO_2_ and F doped FTO NPs with 50 at. % F doping, respectively, which is in accordance with that calculated by XRD patterns. The SnO_2_ NPs (seen in Figure 2b) are polycrystalline and the interplanar spacing is around 3.358 Å, 2.651 Å, 2.377 Å, 1.764 Å, and 1.502 Å corresponding to the lattice plane of (110), (101), (200), (211), and (310), respectively. The element distribution of F, O, and Sn in FTO NPs with 50 at. % F doping is qualitatively exhibited by EELS mapping, as shown in Figure 2d–f. All of the elements are distributed uniformly, particularly fluorine, which proves that F has been successfully doped in SnO_2_ NPs.

### 3.3. Raman Spectra Analysis

**Figure 3:**
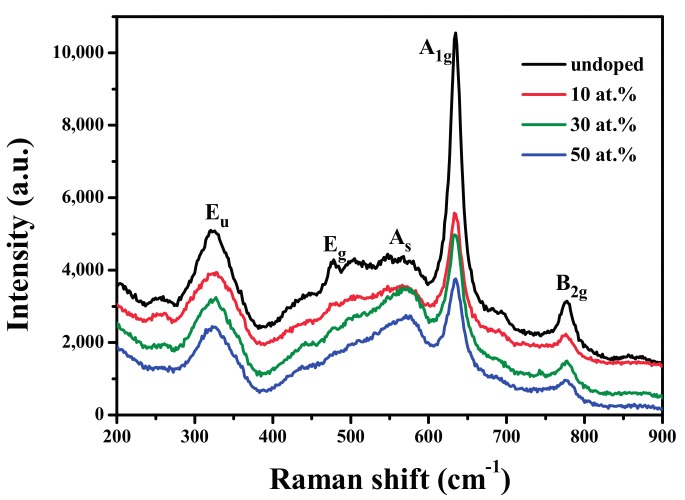
Raman spectra of SnO_2_ and FTO NPs.

Figure 3 shows the evolution of Raman spectra of SnO_2_ and FTO NPs. It can be confirmed from these Raman spectra that SnO_2_ and FTO NPs possess the characteristics of the tetragonal rutile structure, which is in accordance with the XRD and TEM results. The broad peak within the 270–380 cm^−^^1^ region is attributed to the E_u_ mode, indicating the amorphous and nanocrystalline nature of the samples [30,31,32]. The Raman peak at 477 cm^−1^ is corresponding to E_g_ mode, and is related to the vibration of oxygen in the oxygen plane [15,30,31,32]. The A_s_ mode located at around 578 cm^−1^ can be assigned to oxygen vacancies on the grain surface [31,32,33,34]. The increasing intensity of A_s_ mode with the increasing F content is resulted from the higher oxygen vacancy concentration [31,32,33,34]. The detected Raman peaks at 630 cm^−1^ and 773 cm^−1^ correspond to A_1g_ and B_2g_, respectively, and are both related to the expansion and contraction of Sn-O bonds [15,31,32]. The decreasing intensity of these two peaks with the increasing F content in the SnO_2_ lattice might be due to the decreasing grain sizes, because that A_1g_ and B_2g_ modes are sensitive to the grain size [15,30,31,32]. These behaviors might be related to the fact that the F substituting O in the Sn-O bonds changes the local disorder and defects distribution, inhibiting the growth of SnO_2_ NPs [31,32,33,34], as confirmed by TEM and XRD. Therefore, the results provide a good insight into the influence of oxygen vacancies on the Raman behavior in SnO_2_ nanostructured materials.

### 3.4. Optical Properties

**Figure 4:**
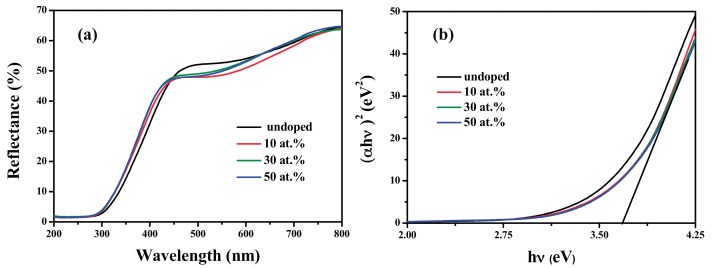
(**a**) Reflectance spectra of SnO_2_ and FTO NPs; (**b**) Plot of (*αhν*)^2^ versus photon energy (*hν*) of SnO_2_ and FTO NPs.

The direct optical band gap (Eg) can be calculated from the absorption coefficient (*α*) and photon energy (*hν*) by the following relation [30]:

(2) $$\alpha h\nu =A{\left(h\nu -\mathrm{Eg}\right)}^{n}$$

where *α* can be calculated by *α* = log (100/R), R is the reflectance coefficient. A is a constant, and *n* depends upon the direct/indirect allowed transition. In this case, *n* equals to 1/2. Then, plot *(αhν)*^2^ versus *hν* and extrapolate the linear portion to the energy axis to obtain Eg [29,30], as shown in Figure 4b. The Eg values of SnO_2_ and FTO NPs are listed in Table 2, and increase from 3.633 eV to 3.684 eV with F doping content, which is higher than the standard value of the bulk SnO_2_ (3.6 eV). This increase can be explained by the Moss-Burstein effect [35,36]. The excessive F^−^ in the n-type SnO_2_ lattice can possibly increase the carrier concentration, which lifts the Fermi level in the conduction band.

### 3.5. XPS Analysis

**Figure 5:**
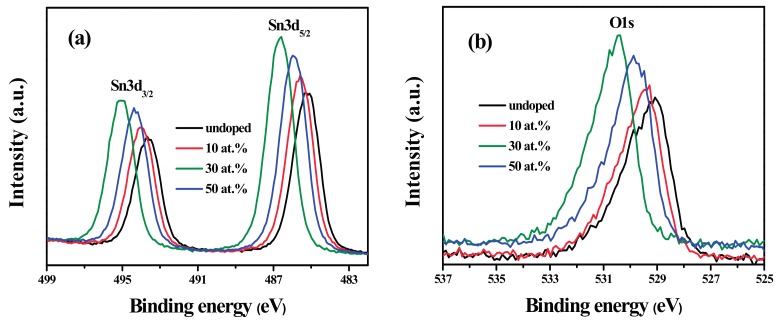
XPS spectra of SnO_2_ and FTO NPs: (**a**) Sn3d; (**b**) O1s.

The typical XPS binding energy spectra for Sn3d and O1s core levels are shown in Figure 5. It can be seen that the Sn3d peaks are symmetrical and separated by 8.4 eV, which is corresponding to the chemical state of Sn^4+^ in SnO_2_. O1s peaks are very asymmetric, indicating that there are more than one kind of chemical states of oxygen [35,36]. In addition, there are small shifts in Sn3d and O1s peaks with the increase of F doping content in Sn3d and O1s peaks. All of these changes are closely related with the incorporation of doped F^−^ at oxygen sites in the SnO_2_ lattice [9,28,36,37,38].

**Figure 6:**
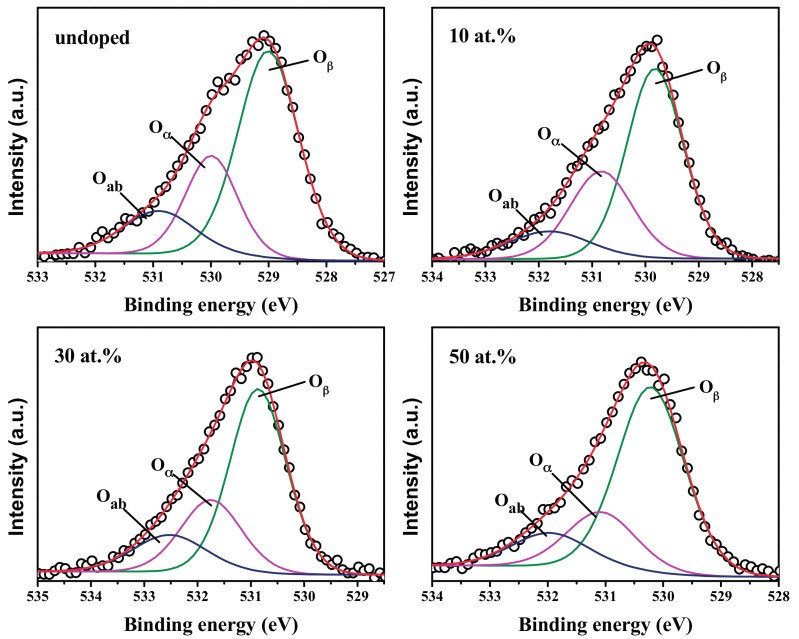
Gaussian deconvoluted X-ray photoelectron spectroscopy (XPS) spectrum of O1s of SnO_2_ and FTO NPs.

Further analysis was conducted as shown in Figure 6. The core level spectra of O1s in SnO_2_ and FTO NPs were fitted utilizing a Gaussion function and classified into three types of oxygen chemical states. O_ab_ corresponds to the hydroxyl groups or the adsorbed oxygen on the particle surface with the highest binding energy [35,36]. The O*_β_* peak with the lowest binding energy is corresponding to the O^2−^ state with the lowest binding energy, which can form [SnO_6_] octahedrons with adjacent Sn^4+^ in the SnO_2_ lattice [35,36]. The O*_α_* peak with the middle binding energy is related to non-stoichiometric oxygen [35,36], which fails to form [SnO_6_] octahedrons.

The relative content and peak position of the three kinds of oxygen chemical states are listed in Table 3. There may exist amount of oxygen defects ($V_{O}^{0}$, $V_{O}^{+}$, and $V_{O}^{++}$) around O*_α_*. When the F doping content is below 30 at. %, the value of O*_α_*% increases to 6.12 at. % with the increasing F doping content. Due to the difference of ion radius and charge numbers between O^2−^ and F^−^, F^−^ can substitute O^2−^ to occupy the regular lattice sites in SnO_2_ [8,15,18]. Therefore, charge imbalance and a lattice distortion are generated, contributing to the formation of point oxygen vacancies [15,16]. When the F doping content reaches to 50 at. %, the value of O*_α_*% decreases considerably to 4.45 at. %. This may due to that F ions occupy oxygen vacancies, which relieve charge imbalance and regulate the lattice order.

### 3.6. Photoluminescence Performance

**Figure 7:**
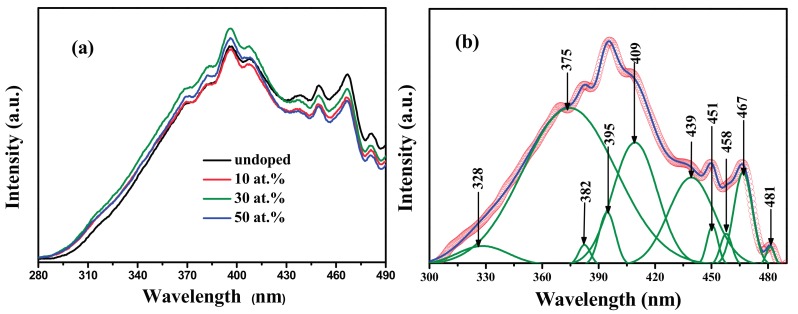
(**a**) Room temperature PL spectra of SnO_2_ and FTO NPs annealed; (**b**) Gaussian deconvoluted PL spectrum of FTO NPs with 50 at. % F doping.

The room-temperature PL spectra under 255 nm wavelength excitation of SnO_2_ and FTO NPs are shown in Figure 7a. It can be seen that all of the samples exhibit a similar type of PL signals with no different peaks, demonstrating that F doping did not cause new PL phenomena.

The Gaussion-Lorentzian function fitting were carried out to the PL spectra of all the samples, and take that of FTO NPs with 50 at. % F doping for example (shown in Figure 7b). It is obvious that the obtained PL spectrum can be deconvoluted into four strong emission peaks along with six weak emission peaks. Among those emission bands, the energy of 328 nm peak (~3.78 eV) is much larger than the band gap (3.684 eV) that is calculated in Table 2, corresponding to a direct recombination of a conduction electron in the Sn4p band and a hole in the O2p valence band. The energy of other peaks is distinctly smaller than the band gap, so it can be deduced that they are induced by defect levels.

**Figure 8:**
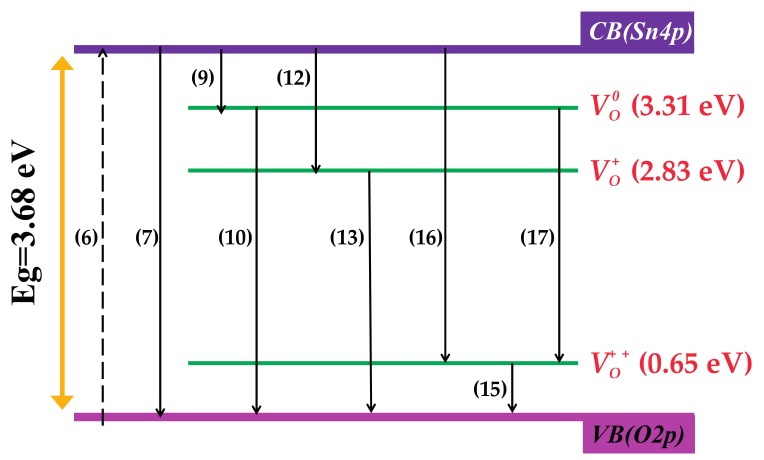
Schematic representation of relaxation process in photoexcited FTO NPs.

It is known that $V_{O}^{0}$ is a shallow donor level that is located near the conduction band [39,40,41]. In this work, we found that the near-band-edge (NBE) emission at 375 nm (~3.31 eV) can be attributed to the electron transition on the $V_{O}^{0}$ level to the valence band. This means that the energy level formed by $V_{O}^{0}$ is located at ~0.37 eV below the bottom of conduction band. This value is nearly the same as that of 0.39 eV, as reported by Mrabet et al. [11]. In addition, the emission peak at 439 nm (2.83 eV) can be assigned to the electron transition from the donor level formed by $V_{O}^{+}$ to the valance band, which is accordance with that of 2.84 eV, as reported by Xu et al. [40]. The level formed by $V_{O}^{++}$ always lies slightly above the valence band [21,22,23,24]. In present work, the emission peaks at 409 nm (3.04 eV) are due to the electron transition from the conduction band to the acceptor level formed by $V_{O}^{++}$, which is accordance with the report from Trani et al. [41]. Meanwhile, the emission peak at 467 nm (2.65 eV) is attributed to the electron transition from the donor level formed by $V_{O}^{0}$ to the acceptor level formed by $V_{O}^{++}$. According to the analysis of PL spectra, a schematic band diagram of SnO_2_ and FTO NPs with defect levels formed by oxygen vacancies is presented in Figure 8. The energy levels formed by $V_{O}^{0}$ and $V_{O}^{+}$ are located at 0.37 eV and 0.85 eV below the conduction band (Sn4p), respectively, while the energy level formed by $V_{O}^{++}$ is located at 0.65 eV above the valence band (O2p).

### 3.7. Investigation of Photoluminescence Mechanism

The intensity variation of PL property with F doping content can be explained by two main factors: one is the content of oxygen vacancies mentioned at XPS analysis, and the other one is the changes of the quantity of different types of oxygen defects, because that $V_{O}^{0}$, $V_{O}^{+}$, and $V_{O}^{++}$ can change to each other with *e*^−^ [30,39,40]. In our view, F^−^ entering into the SnO_2_ lattice can adjust the band structure of SnO_2_ crystallite and then affect the luminescence performance. To the fact that the donor character F^−^ is more pronounced than that of O^2−^ in SnO_2_ matrix, F^−^ may substitute O^2−^ and generate $F_{O}^{+}$ and one extra *e*^−^. Subsequently, *e*^−^ can combine with $V_{O}^{+}$ or $V_{O}^{++}$ to generate $V_{O}^{0}$ or $V_{O}^{+}$ to ensure charge balance, as shown by Equations (3)–(5).

(3) $$O_{O}\to F_{O}^{+}+e^{-}$$

(4) $$e^{-}+V_{O}^{++}\to V_{O}^{+}$$

(5) $$e^{-}+V_{O}^{+}\to V_{O}^{0}$$

The intensity of 375 nm peak increases with the F doping content and may be attributed to the increase of the amount of oxygen vacancies, as well as $V_{O}^{+}$ turning into $V_{O}^{0}$. Well, the decrease of 467 nm peak intensity with F doping is due to that $V_{O}^{++}$ turning into $V_{O}^{+}$ reduces the amount of $V_{O}^{++}$ (Equation (4)). The interconversion among $V_{O}^{0}$, $V_{O}^{+}$ and $V_{O}^{++}$ cooperated with different F doping content has accomplished in preparation programs. No more changes of $V_{O}^{0}$, $V_{O}^{+}$, and $V_{O}^{++}$ quantities would occur when PL property was tested.

Based on the fact that the photoluminescence essence is the separation and recombination of electron-hole pairs, an assumption model about the luminescence process of FTO NPs proposed as follows (*p_xx_* is neutral granule; *hν_e_* is the incident photons; *hν_others_* is the energy of other recombination processes, probably corresponding to weak emission peaks on PL spectrum; *Q* represents non-radiative energy), and the formulas associated to the luminescence processes are also given in Figure 8.

The separation and recombination of electron-hole pairs between conduction band and valence band (*hν_Eg_*):

(6) $$p_{VB}+h\nu_{e}\to h_{VB}^{+}+e_{VB\to CB}^{-}$$

(7) $$h_{VB}^{+}+e_{CB\to VB}^{-}\to P_{VB}+h\nu_{Eg}$$

The separation and recombination of electron-hole pairs between $V_{O}^{0}$ and valence band (*hν*_1_):

(8) $$p_{V_{O}^{0}}\to h_{V_{O}^{0}}^{+}+e_{V_{O}^{0}\to VB}^{-}$$

(9) $$h_{V_{O}^{0}}^{+}+e_{CB\to V_{O}^{0}}^{-}\to p_{V_{O}^{0}}+h\nu_{\mathrm{others}}/Q$$

(10) $$h_{VB}^{+}+e_{V_{O}^{0}\to VB}^{-}\to h\nu_{1}$$

The separation and recombination of electron-hole pairs between conduction band and $V_{O}^{+}$ (*hν*_3_):

(11) $$p_{V_{O}^{+}}\to h_{V_{O}^{+}}^{+}+e_{V_{O}^{+}\to VB}^{-}$$

(12) $$h_{V_{O}^{+}}^{+}+e_{VB\to V_{O}^{+}}^{-}\to p_{V_{O}^{+}}+h\nu_{\mathrm{others}}/Q$$

(13) $$h_{VB}^{+}+e_{V_{O}^{+}\to VB}^{-}\to P_{VB}+h\nu_{2}$$

The separation and recombination of electron-hole pairs between conduction band and $V_{O}^{++}$ (*hν*_2_):

(14) $$p_{V_{O}^{++}}\to h_{V_{O}^{++}}^{+}+e_{V_{O}^{++}\to VB}^{-}$$

(15) $$h_{VB}^{+}+e_{V_{O}^{++}\to VB}^{-}\to p_{VB}+h\nu_{\mathrm{others}}/Q$$

(16) $$h^{+}{}_{V_{O}{}^{++}}+e^{-}{}_{CB\to V_{0}{}^{++}}\to P_{V_{O}{}^{++}}+hv_{2}$$

The separation and recombination of electron-hole pairs between $V_{O}^{0}$ and $V_{O}^{+}$ (*hν*_4_):

(17) $$e_{V_{O}^{0}\to V_{O}^{++}}^{-}+h^{+}{}_{V_{O}{}^{++}}\to P_{V_{O}{}^{++}}+hv_{4}$$

## 4. Conclusions

All of the SnO_2_ and FTO NPs prepared by hydrothermal method with subsequent annealing at 500 °C possess polycrystalline tetragonal rutile structure and the high UV-violet emissions, which can be one promising candidate of short wavelength optoelectronic materials. All PL spectra of SnO_2_ and FTO NPs have quite similar shapes and positions of PL peaks, but different relative intensity. The doping element F^−^ can substitute O^2−^ into the SnO_2_ lattice, generating $F_{O}^{+}$ and one extra *e*^−^. The extra *e*^−^ can combine with $V_{O}^{+}$ or $V_{O}^{++}$ to generate $V_{O}^{0}$ or $V_{O}^{+}$ to ensure charge balance. The energy level that is formed by $V_{O}^{0}$ and $V_{O}^{+}$ is located at 0.37 eV and 0.65 eV below the bottom of conduction band, respectively, while that formed by $V_{O}^{++}$ is located at 0.65 eV above the valence band.

## Figures and Tables

**Table 1:** Lattice information calculated from XRD of SnO_2_ and FTO NPs.

Samples	D-Spacing			Lattice Parameters			D_aver_ (nm)
	d_110_ (Å)	d_101_ (Å)	d_211_ (Å)	a (Å)	c (Å)	V (Å^3^)	
PDF#71-0652	3.350	2.644	1.764	4.738	3.187	71.5	—
Undoped	3.351	2.647	1.763	4.730	3.194	71.44	20.18
10 at. %	3.351	2.650	1.765	4.731	3.198	71.60	18.19
30 at. %	3.356	2.653	1.763	4.718	3.208	71.41	17.08
50 at. %	3.343	2.647	1.761	4.718	3.197	71.16	16.59

**Table 2:** The optical band gap of SnO_2_ and FTO NPs.

Samples	Undoped	10 at. %	30 at. %	50 at. %
Eg (eV)	3.633	3.681	3.682	3.684

**Table 3:** Binding energy, FWHM and the relative content of Gaussian peaks and relative content of O1s of SnO_2_ and FTO NPs.

Sample	O1s				Relative Content of O*_α_*
	Relative Content	Gaussian Peak	Peak Position (eV)	Area (%)	
Undoped	16.3 at. %	O_ab_	530.9	14.85	4.04 at. %
		O*_α_*	529.99	24.78	
		O_β_	529.01	60.37	
10 at. %	18.55 at. %	O_ab_	531.3	12.2	5.5 at. %
		O_α_	530.33	29.66	
		O_β_	529.32	58.13	
30 at. %	24.36 at. %	O_ab_	532.02	15.46	6.12 at. %
		O*_α_*	531.25	25.12	
		O_β_	530.37	59.42	
50 at. %	21.55 at. %	O_ab_	531.46	13.64	4.45 at. %
		O*_α_*	530.58	20.67	
		O_β_	529.71	65.69	
